# Mice Lacking Alkbh1 Display Sex-Ratio Distortion and Unilateral Eye Defects

**DOI:** 10.1371/journal.pone.0013827

**Published:** 2010-11-03

**Authors:** Line M. Nordstrand, Jessica Svärd, Elisabeth Larsen, Anja Nilsen, Rune Ougland, Kari Furu, Guro F. Lien, Torbjørn Rognes, Satoshi H. Namekawa, Jeannie T. Lee, Arne Klungland

**Affiliations:** 1 Centre for Molecular Biology and Neuroscience, Institute of Medical Microbiology, Oslo University Hospital and University of Oslo, Oslo, Norway; 2 Department of Molecular Biology, Massachusetts General Hospital, Boston, Massachusetts, United States of America; 3 Department of Informatics, University of Oslo, Oslo, Norway; 4 Institute of Basic Medical Sciences, University of Oslo, Oslo, Norway; 5 Howard Hughes Medical Institute, Massachusetts General Hospital, Boston, Massachusetts, United States of America; City of Hope National Medical Center, United States of America

## Abstract

**Background:**

*Eschericia coli* AlkB is a 2-oxoglutarate- and iron-dependent dioxygenase that reverses alkylated DNA damage by oxidative demethylation. Mouse AlkB homolog 1 (Alkbh1) is one of eight members of the newly discovered family of mammalian dioxygenases.

**Methods and Findings:**

In the present study we show non-Mendelian inheritance of the *Alkbh1* targeted allele in mice. Both *Alkbh1^−/−^* and heterozygous *Alkbh1^+/−^* offspring are born at a greatly reduced frequency. Additionally, the sex-ratio is considerably skewed against female offspring, with one female born for every three to four males. Most mechanisms that cause segregation distortion, act in the male gametes and affect male fertility. The skewing of the sexes appears to be of paternal origin, and might be set in the pachythene stage of meiosis during spermatogenesis, in which *Alkbh1* is upregulated more than 10-fold. In testes, apoptotic spermatids were revealed in 5–10% of the tubules in *Alkbh1^−/−^* adults. The deficiency of *Alkbh1* also causes misexpression of *Bmp2*, *4* and *7* at E11.5 during embryonic development. This is consistent with the incompletely penetrant phenotypes observed, particularly recurrent unilateral eye defects and craniofacial malformations.

**Conclusions:**

Genetic and phenotypic assessment suggests that Alkbh1 mediates gene regulation in spermatogenesis, and that Alkbh1 is essential for normal sex-ratio distribution and embryonic development in mice.

## Introduction

The *Eschericia coli* (*E. coli*) DNA repair enzyme AlkB demethylates e.g. 1-methyladenine (1-meA) to adenine – generating succinate and formaldehyde – in the presence of iron as cofactor and 2-oxoglutarate as cosubstrate [1], [2]. To date, eight AlkB homologs have been identified in the mammalian genome [3]. Except for Alkbh5, all the remaining proteins have been identified throughout the animal kingdom, suggesting fundamental roles in biological processes [4]. Two of these homologs, ALKBH2 and ALKBH3 in humans (Alkbh2 and Alkbh3 in mice), are similar to *E. coli* AlkB in that they efficiently repair damaged nucleic acids in the presence of iron and 2-oxoglutarate *in vitro* [5]–[9]. In mice, Alkbh2 is the major, probably only, dioxygenase that repairs 1-meA DNA *in vivo* and mice lacking Alkbh2 accumulate 1-meA in the genome during ageing [10]. This year, two groups reported that Alkbh8 is a tRNA methyltransferase required for the final step in the biogenesis of mcm^5^U [11], [12]. ALKBH8 plays important roles in the survival and progression of human bladder cancer both *in vitro* and *in vivo* [13]. A likely ninth AlkB homolog, the obesity-associated Fto protein, was shown to have potential to demethylate 3-methylthymine (3-meT) [14], [15]. Crystal structure of the FTO protein recently confirmed this, and indicated that single-stranded RNA is the primary substrate of FTO [16]. Similarly, recombinant truncated Alkbh1 enzyme may demethylate 3-methylcytosine *in vitro* [17], but it remains unclear whether this activity is physiologically relevant.

All eight mammalian AlkB homologs contain the conserved iron- and 2-oxoglutarate dioxygenase domain. However, the region of *E. coli* AlkB that interacts with the nucleic acid substrate, the N-terminal nucleotide recognition lid, does not share sequence similarity with the mammalian homologs. Therefore, one cannot exclude the possibility that the targets of such proteins are not nucleic acids, but other macromolecules such as proteins. Since JmjC histone demethylases remove methyl groups from histones using the same mechanism as *E. coli* AlkB, it has been suggested that Alkbh1, 4 and 7 might be involved in histone/protein demethylation [18], [19]. However, for Alkbh1 we, and others, have been unable to identify DNA/histone demethylation activity [6], [7], [20], [21]. In 2008 a paper on Alkbh1 was published by Pan et al, where a gene-targeting study in mice showed that Alkbh1 localizes to nuclear euchromatin and functions in epigenetic regulation of gene expression [20]. Their study demonstrated impaired placental trophoblast lineage differentiation in *Alkbh1* ^−/−^ mice, and a strong interaction of Alkbh1 with Mrj, an essential placental gene that mediates gene repression by recruitment of class II histone deacetylases (HDAC) [20].

In the present study we attempt to elucidate the role of Alkbh1 by targeted deletion in C57/BL6 mice. We demonstrate that *Alkbh1* deficiency in mice results in apoptosis in adult testes and sex-ratio distortion of offspring, most likely caused by defects in the pachytene stage during spermatogenesis. An incompletely penetrant phenotype apparent during embryonic development is consistent with *Bmp2*, *4* and *7* misexpression. Although many mechanistic aspects of Alkbh1 function remain to be revealed, these results show that Alkbh1 is crucial for normal embryonic development and viability in mice, and plays an important role during spermatogenesis.

## Materials and Methods

### Generation of *Alkbh1* Targeted Mice

A specific 360-bp murine probe of exon 6 in the *Alkbh1* gene was amplified from mouse genomic DNA by polymerase chain reaction (PCR) and used to screen a 129 SvJ mouse genomic library (Stratagene). To generate the targeting construct, we subcloned fragments from a ∼14-kb genomic clone on both sides of neomycin *(neo)* in the pGT-N38 vector (New England Biolabs). Homologous arms consisting of a 3.0-kb MfeI/HindIII fragment and a 3.7-kb BsrGI fragment facilitated removal of a 3.8-kb HindIII/BsrGI fragment including exon 6 and replacement with the *neo* cassette. The targeting construct was electroporated into 129 SvJ embryonic stem (ES) cells, and transfectants were selected in geneticin (G418) and expanded for further analysis. Chimaeric mice were produced by microinjection of one targeted ES cell clone with normal karyotype into C57/BL6 blastocysts at embryonic day 3.5 (E3.5). We verified germline transmission of the targeted allele by Southern-blot analysis of ScaI-digested genomic DNA on the 5′ end and PCR analysis on the 3′ end. 5′ and 3′ homologous recombination in the F_1_ generation were confirmed by PCR analysis. Heterozygous males were backcrossed for three generations onto C57/BL6 females. All mouse experiments were approved by the Norwegian Animal Research Authority (Ref. nr. 08/9940) and done in accordance with institutional guidelines at the Centre for Comparative Medicine at Oslo University Hospital. Animal work was conducted in accordance with the rules and regulations of the Federation of European Laboratory Animal Science Association's (FELASA).

### Genotyping

For *Alkbh1* genotyping, ear-clip samples were degraded by incubation in PBND buffer (50 mM KCl, 10 mM Tris-HCl pH 8.3, 2.5 mM MgCl_2_-6H_2_O, 0.1 mg/ml gelatin, 0.45% v/v NP40, 0.45% v/v Tween 20) and 0.5 mg/ml proteinase K at 55°C over night. Samples were heated to 95°C for 10 min to inactivate proteinase K, and PCR amplified for 35 cycles with an annealing temperature of 60°C (see primers below). For sex genotyping of embryos, a small piece of tissue was obtained from the embryosac or -tail and washed three times in PBS to eliminate maternal contamination. The tissue was degraded by a 3-hour incubation, and subsequently treated as above. PCR analysis of *Sry* (Y-linked gene) was performed to determine maleness and *Rapsn* was used as an autosomal, internal control as described (Mouse Phenotypes, a Handbook of Mutatation Analysis, Cold Spring Harbor laboratory press, Chapter 3, page 40, 2005).

Primers wild-type allele (WT): 5′-AGTTATCAGGGCCATCCAGGGAGGT-3′

5′-AACTGAGAGGTACAGGGAAGCATAA-3′

Primers targeted allele (KO): 5′-GCTTGCCGAATATCATGGTG-3′

5′-AACTGAGAGGTACAGGGAAGCATAA-3′

### Whole-Mount *In Situ* Hybridization

We carried out whole-mount *in situ* hybridization on E9.5 to E12.5 embryos fixed in paraformaldehyde as described (Henrique et al. 1995). Mouse antisense and sense (control) RNA probes were prepared using DIG RNA labeling mix (Roche) together with T3 or Sp6 and T7 RNA polymerases (Roche). Templates for the labeling reaction were PCR products amplified from full-length mouse cDNA with T3, Sp6 or T7 promoters added to the PCR primers. For *Alkbh1* the template contained 465-bp of exon 6, for *Bmp2* 519-bp of exon 2–3 and for *Bmp7* 559-bp of exon 2–5. For *Bmp4*, linearized pSP72 plasmid with a 1550-bp insert was used as template. Embryos were examined on a SMZ1500 microscope (Nikon).

### Quantitative Real-Time PCR (qPCR) Analysis

Total RNA was isolated from embryos, organs and germ cells using the Fast RNA Pro Green Kit (MP Biomedicals) according to the manufacturers protocol. Any DNA remnants were removed using TURBO DNase (Ambion) and cDNA was made using High Capacity cDNA Reverse Transcription Kit (Applied Biosystems). The quantitative PCR reactions were carried out on a StepOnePlus or 7500 Fast instrument using 50 ng cDNA, TaqMan® Fast Universal PCR Master Mix and appropriate TaqMan primers and probes (all from Applied Biosystems). Pre-designed primers and probes were used both for the target genes (*Alkbh1, Vav2, Mapk8, Ccdc80, Rest, and Hif1a*) and endogenous controls (*Gapdh, 18s* and *β-actin*). All samples were run in triplicates and with one technical parallel (2 runs per sample). The relative quantity was calculated using the equation RQ = 2^−ΔΔCT^, where RQ is the relative quantity of the target gene. ΔΔCT is the difference in CT-value between the target gene and the endogenous control minus the difference in CT-values between the reference gene and the endogenous control.

### STAPUT Isolation of Testicular Cells

Male germ cells were isolated from testes using an adapted version of the STAPUT method [22]. Pachytene cells and round spermatids were isolated from six 12-week old males, while a total of sixty 10-day old males were sacrificed for the isolation of type A and type B spermatogonia. The testes were put in ice cold DMEM medium containing antibiotics and then carefully detunicated. The tubules were treated with DNaseΙ, collagenase, trypsin and hyaluronidase (all from Sigma-Aldrich) at 34°C to remove connective tissue and somatic cells, yielding a cell suspension of germinal cells in DMEM containing 0.5% BSA. The cell suspension was loaded into the cell loading chamber of the STAPUT apparatus and separated by sedimentation velocity at unit gravity in a 2–4% w/v BSA gradient in DMEM medium at 4°C for 2.5 hours. After sedimentation, 10 ml fractions were collected and checked under the microscope. Fractions containing pure germ cells were pooled and the cell number counted in a *Countess*® Automated Cell Counter (Invitrogen). Cells were spun down and the pellet was snap frozen in liquid nitrogen before placed in −70°C. An aliquot of isolated cells was fixed on SuperFrost Plus slides (VWR) using Cell Adherence Solution (Crystalgen, Lot no 425081) for microscopic analysis of purity.

### TUNEL Assay of Testes

We fixed testes from 3- and 9-month old animals in neutral-buffered formalin, progressively dehydrated them in a graded ethanol series, and embedded them in paraffin. Sections (4-µm) were deparaffinized and treated with proteinase K for 15 min and quenched in 3% hydrogen peroxide in PBS for 5 min at room temperature. Subsequently, nuclear staining in apoptotic cells was detected using ApopTag kit (Chemicon, http://www.chemicon.com) according to the manufacturers instruction. Sections were analysed on an Axioplan 2 microscope (Zeiss).

### Immunofluorescent Staining of Testicular Cells

Testicular cells from 12-month old males were spread on SuperFrost Plus slides (VWR), progressively dehydrated in a graded ethanol series and dried completely. Slides were washed in 1× PBS and fixated in 4% PFA in PBS. Slides were blocked in 5% serum in PBS for 1 hour at room temperature and incubated with primary antibodies overnight at 4°C prior to detection with secondary antibodies. Primary antibodies used were rabbit anti-MacroH2A (1∶500, Upstate) and mouse anti-FK2 (1∶5000, Biomol). Secondary antibodies used were goat anti-rabbit Alexa Fluor 488 (green dye) (Invitrogen) and goat anti-mouse Alexa Fluor 594 (red dye) (Invitrogen), respectively. Single Z-sections were captured by Axioplan 2 microscope (Zeiss).

### DNA Microarray Analysis

High quality of total RNA extracted from adult testes was verified on Agilent Bioanalyzer 2100 (RIN value between 9.8 and 10.0). 15 µg of biotinylated and fragmented cRNA was then hybridized onto the GeneChip Mouse Genome 430 2.0 Array (Affymetrix) according to manufacturers protocols (Affymetrix). QCs including scale factor, background, noise, spikes and RNA degradation were checked and validated using the yaqcaffy library (http://www.bioconductor.org/packages/2.3/%20bioc/vignettes/yaqcaffy/inst/doc/yaqcaffy.pdf).

Affymetrix raw data was generated with GCOS 1.4 (GeneChip Operating Software, Affymetrix), and the signal intensities of each probe set were normalized with the RMA (Robust Microarray Anlaysis) algorithm. To find differentially expressed genes, t-test with randomized variance was used as statistical test and the cut-off (p-value) was set to 0.05 with a FDR correction. Class comparison analysis was used to identify interesting genes. The signal in one group was always (i.e. for all the triplicate) higher or lower compared to the other group. Fold change for all the genes that passed the above criteria was computed and only the genes with ≥2-fold change were studied. The heatmap was generated using the GeneSpring GX 10 demoversion (Agilent). All data is MIAME compliant and the raw data has been deposited in a MIAME compliant database, the accession number is GSE22073.

### Skeletal Staining

For skeletal analysis, skin and internal viscera of E18.5 embryos and newborn mice were removed. We then fixed the animals overnight in 95% ethanol and carried out Alcian blue 8GX (Sigma) and Alizarin red S (Sigma) staining of cartilage and bone, respectively, as described (Manipulating the Mouse Embryo, 3^rd^ edition, Cold Spring Harbor laboratory press, Chapter 16, Protocol 22, pages 699–700, 2003). The skeletons were photographed with a Nikon D80 camera.

### Histological Analysis of Eyes

We fixed adult eyes in neutral-buffered formalin or paraformaldehyde added 20% absolute alcohol for 24 hours, progressively dehydrated them in a graded ethanol series, and embedded them in paraffin. Sections (4-µm) were deparaffinized, rehydrated and stained with hematoxylin and eosin. Sections were analysed on an AxioCam HRc microscope (Zeiss).

## Results

### Deletion of *Alkbh1* in Embryonic Stem Cells and Mice

**Figure 1:**
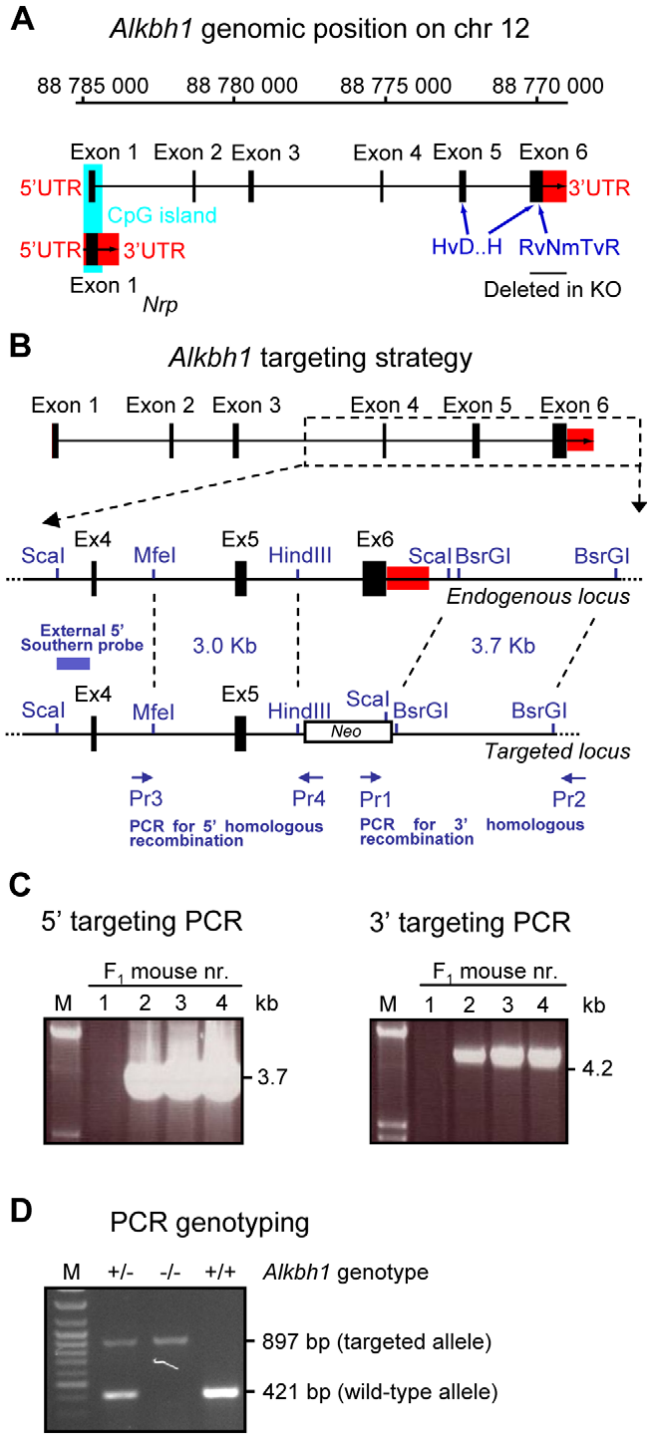
Targeted disruption of *Alkbh1* in embryonic stem cells and mice. (A) Schematic representation of the genomic region harboring the *Alkbh1* gene. Exon 6 is replaced by neomycin, thus maintaining the overlapping *Nrp* gene, and removing the conserved 2-oxoglutarate interaction domain (RvNmTvR) and parts of the iron-binding cluster (HvD…H) essential for enzymatic activity. A CpG island of 550 bp, shown in blue, is surrounding exon 1 (criteria used: Island size >200, GC Percent >50.0, Obs/Exp >0.6). The *Nrp* gene displays an overlap with exon 1 and is encoded as a forward frameshift to *Alkbh1* in the mouse. The 5′ and 3′ UTRs of *Alkbh1* and *Nrp* are shown in red. Coding exons are shown as black boxes. (B) Overview of the *Alkbh1* targeting strategy. Upper, schematic map of the genomic *Alkbh1* locus. Dashed lines point out the region used for homologous recombination. Middle, partial resctriction map of the endogenous *Alkbh1* locus participating in homologous recombination. Bottom, the targeted *Alkbh1* locus after correct integration of genomic fragments consisting of a 3.0-kb MfeI/HindIII fragment and a 3.7-kb BsrGI fragment on both sides of *Neo*, thereby replacing a 3.8-kb HindIII/BsrGI fragment including exon 6 with *Neo*. (C) PCR analysis for verification of 5′ and 3′ homologous recombination in the F_1_ generation. The 3.7-kb 5′ targeted band (Pr3, Pr4) and the 4.2-kb 3′ targeted band (Pr1, Pr2) is present in F_1_ mouse nr. 2, 3 and 4. M is the DNA marker. (D) PCR genotyping of the *Alkbh1* allele. The 421-bp wild-type band (WT) and the 897-bp targeted band (KO) is shown. M is the DNA marker.

To gain more insight into the role of the Alkbh1 dioxygenase we have generated mice lacking *Alkbh1*. Alkbh1 was the first mammalian AlkB homolog to be identified [23], and is the AlkB homolog most similar in sequence to *Eschericia coli (E. coli)* AlkB. The region of greatest similarity includes 107 amino acids, 37% of which are identical between the *E. coli* and mouse Alkbh1. The conserved RvNmTvR and HvD…H motifs of the 2-oxoglutarate and iron binding sites, respectively, are also present in both proteins. The conserved domains of Alkbh1 are encoded by exon 5 and 6 at the 3′ end of the mouse *Alkbh1* gene. To fully eliminate the activity of Alkbh1 and keep the overlapping *Nrp* gene intact, we substituted exon 6 with a neomycin-resistance gene cassette by homologous recombination in mouse embryonic stem cells (Figs. 1A–D). The expression of the *Nrp* gene was confirmed by qPCR (data not shown).

### Expression Analysis of *Alkbh1* in Embryos, Organs and Male Germ Cells

**Figure 2:**
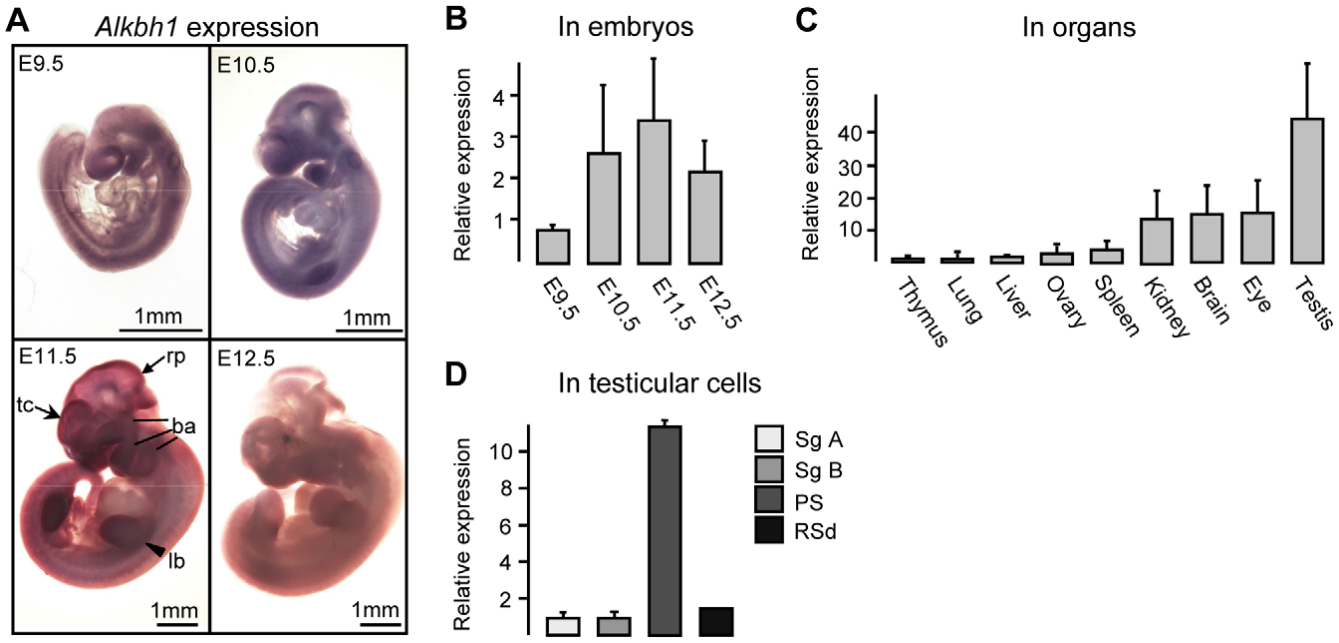
Expression of *Alkbh1* in embryos, organs and male germ cells. (A) Whole-mount *in situ* hybridization of *Alkbh1* between E9.5 and E12.5, side view. Peak expression is revealed at E11.5 in the telencephalon (tc) and frontonasal process, the maxillary and mandibular and hyoid arches (ba), the upper and lower limb buds (lb), and the midbrain and rhombomere 1 (r1) roof plates (rp). (B) Expression of *Alkbh1* between E9.5 and E12.5 by qPCR in RNA extracted from three - eight whole embryos. Peak expression at E11.5 is confirmed. Reference sample, E9.5 (RQ = 1.00); endogenous control, *Gapdh*. (C) Expression of *Alkbh1* in mouse organs by qPCR in RNA extracted from three - five 12-week old mice. Peak expression is shown in testis. Reference sample, thymus (RQ = 1.00); endogenous control, *18s*. (D) Expression of *Alkbh1* at different stages of spermatogenesis. Male germ cells from C57/BL6 mice were STAPUT sorted into type A spermatogonia (Sg A), type B spermatogonia (Sg B), pachytene spermatocytes (PS) and round spermatids (RSd), and analysed by qPCR after RNA extraction from the purified cell populations. Reference sample, type A spermatogonia (RQ = 1.00); endogenous control, *β-actin.*

The expression pattern of *Alkbh1* was analysed in embryos at different stages by whole-mount *in situ* hybridization (Fig. 2A) and by qPCR (Fig. 2B). Weak expression of *Alkbh1* was observed throughout the embryo at E8.5 (data not shown). As the cells migrate and differentiate during organogenesis the expression becomes more specific, and *Alkbh1* was detected in the spinal cord, forebrain and branchial arches at E9.5, and also in limb buds at E10.5 (Fig. 2A). Peak expression was detected at E11.5 in the frontonasal process including telencephalon (tc), maxillary, mandibular and hyoid arches (ba), upper and lower limb buds (lb), and midbrain and rhombomere 1 (r1) roof plates (rp) (Fig. 2A). *Alkbh1* expression decreased considerably from E11.5 to E12.5 (Fig. 2A–B). In adult organs, *Alkbh1* was highly expressed in testis (RQ = 44.0), with slightly lower expression in eye, brain and kidney (RQ = 16.0, 15.4, 14.4) (Fig. 2C). Moreover, the expression of *Alkbh1* was studied at different stages during spermatogenesis, and was found to be significantly elevated in the pachytene spermatocytes (PS) (RQ = 11.3) compared with spermatogonia A and B (Sg A, Sg B) and round spermatids (RSd) (RQ = 1.7) (Fig. 2D). This is the third stage of the prophase of meiosis I, in which synapsis is completed and homologous recombination occurs. Thus, Alkbh1 may have considerable potential for gene-function in embryonic development and in the pachytene stage during spermatogenesis.

### Non-Mendelian Inheritance and Sex-Ratio Distortion in *Alkbh1* Targeted Mice

**Figure 3:**
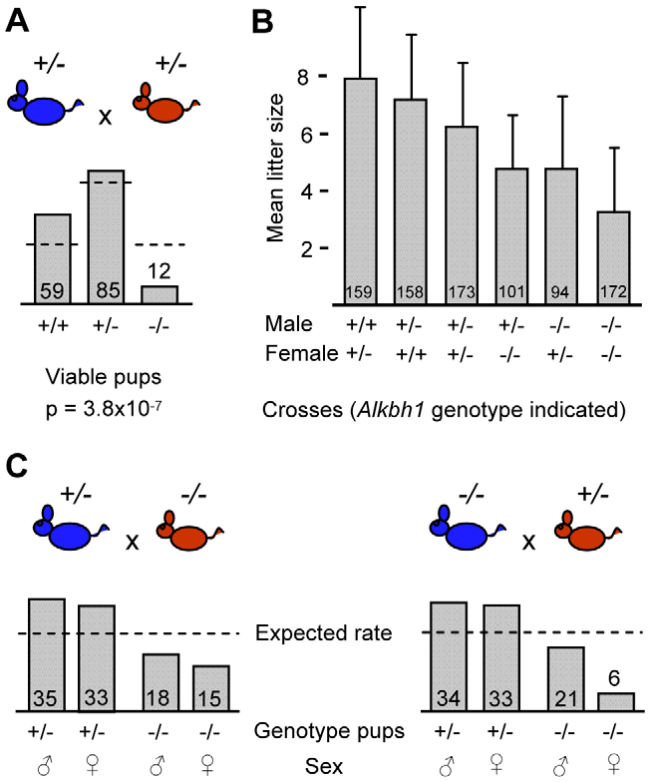
Non-Mendelian inheritance and sex-ratio distortion in *Alkbh1* targeted mice. (A) Offspring distribution of different genotypes at 1-month after crosses between heterozygous males (blue) and females (red) is shown (+ indicates wild-type *Alkbh1* allele and – indicates targeted *Alkbh1* allele). Mean number of pups per cross from 25 litters are represented. Dashed lines represent expected Mendelian distribution, and the χ^2^-test was used to determine significance. (B) Average litter sizes from all crosses at 1-month of age are presented on the y-axis, while *Alkbh1* genotype of males and females used in the different crosses are indicated on the x-axis. The corresponding number of pups from more than 20 litters per cross are shown on the bars. (C) Left panel, crosses between heterozygous males (blue) and homozygous females (red). Right panel, crosses between homozygous males (blue) and heterozygous females (red). Offspring distribution of different *Alkbh1* genotypes at 1-month is shown. Mean number of pups per cross, calculated from 21 litters in the left panel and 20 litters in the right panel, are shown. Dashed lines represent expected sex-ratio distribution.

Mendelian inheritance, in which each parent contributes one of two possible alleles for a given trait, has a characteristic ratio of 1∶2∶1 after heterozygous crosses. Initial crosses of mice carrying either one or two targeted *Alkbh1* loci revealed non-Mendelian distribution. Therefore, we carried out extensive breeding analysis and genotyped more than 1400 *Alkbh1* mutant mice and embryos (Fig. 3). Following heterozygous breedings, the survival of *Alkbh1^−/−^* pups after 1 month was only 20% compared with wild-type littermates (Fig. 3A). In addition, the frequency of viable *Alkbh1^+/−^* mice was only 60% of the expected rate (Fig. 3A). The non-Mendelian distribution was clearly significant with a p-value of 3.8×10^−7^ (χ^2^-test). A similar pattern was observed in *Alkbh1^+/−^* male x *Alkbh1^−/−^* female crosses, p = 5×10^−4^ (χ^2^-test) and *Alkbh1^−/−^* male x *Alkbh1^+/−^* female crosses, p = 3.7×10^−5^ (χ^2^-test) (Fig. 3C). In general, the average litter size decreased as the number of targeted alleles in the parental generation increased (Fig. 3B). The mean litter size was 9.2 for wild-type crosses, 6.2 for heterozygous crosses and 3.2 for homozygous crosses (Fig. 3B). Notably, paternal inheritance of the targeted allele seemed to be more critical than maternal transmission for the survival of offspring. Another evident phenotype was the growth retardation observed in viable *Alkbh1^−/−^* mice compared with wild-type littermates (Fig. S1).

One process of non-Mendelian inheritance is segregation distortion. There are a number of mechanisms that can cause segregation distortion, and both autosomal sex-ratio distortion as well as segregation distortion of the sex chromosomes exist [24]. In *Alkbh1^+/−^* male x *Alkbh1^−/−^* female crosses, the ratio of female to male homozygous offspring at 1 month was approximately 1∶1 (Fig. 3C, left panel). In *Alkbh1^−/−^* male x *Alkbh1^+/−^* female crosses, the ratio of homozygous *Alkbh1^−/−^* pups was significantly skewed against females, with one female born for every three to four males (Fig. 3C, right panel). The survival of *Alkbh1^−/−^* male pups was 60% compared with *Alkbh1^+/−^* pups, whereas the proportion of viable *Alkbh1^−/−^* female pups was only 18%, p = 7.1×10^−5^ (χ^2^-test) (Fig. 3C, right panel). Following heterozygous crosses, the survival of *Alkbh1^−/−^* offspring was significantly reduced, 30% of the males and just 10% of the females survived compared with wild-type littermates, p = 1.4×10^−6^ (χ^2^-test) (data not shown). A sex-ratio distortion was also seen in mid-stage *Alkbh1^−/−^* embryos (E10–E12.5) after heterozygous breedings (17 litters), with 89% male and 60% female embryos present compared with wild-type embryos (data not shown).

### Spermatogenic Defects in *Alkbh1* Deficient Testis

**Figure 4:**
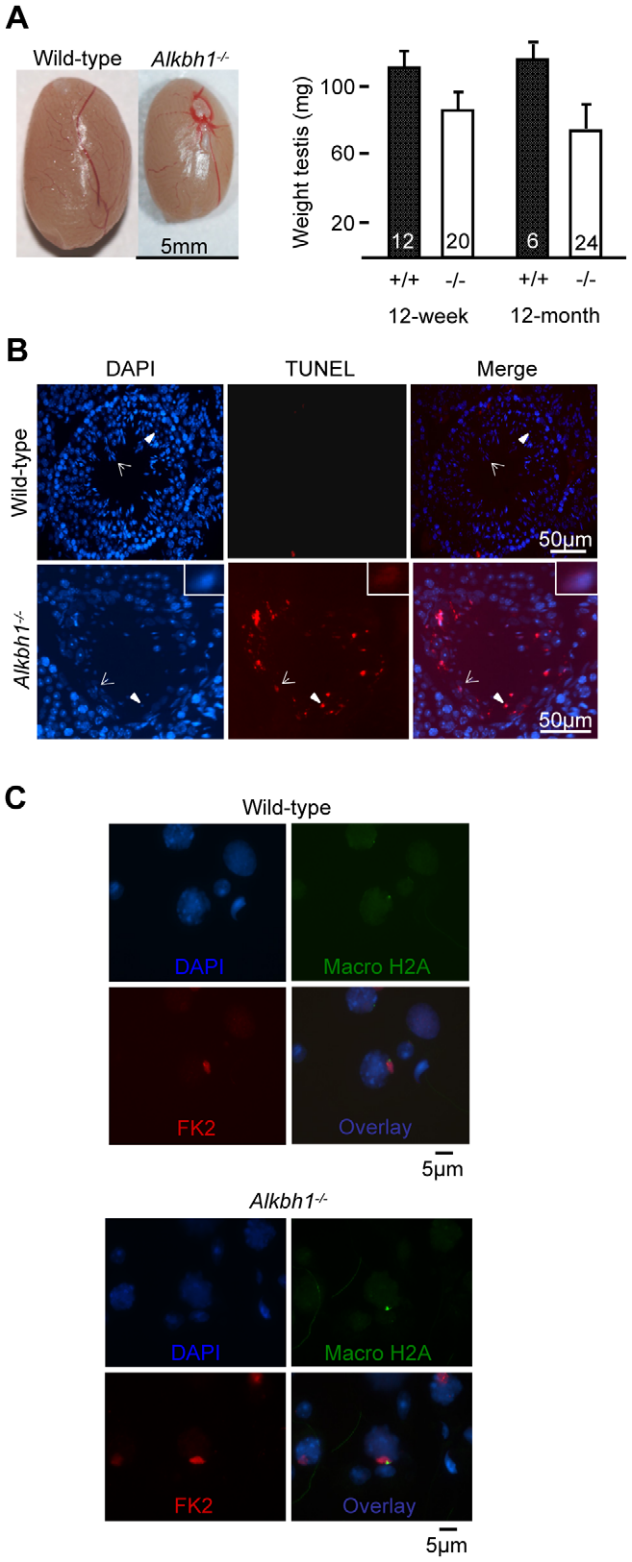
Spermatogenic defects in *Alkbh1* deficient testis. (A) Left panel, representative testis from 12-month old wild-type and *Alkbh1^−/−^* males. Right panel, average testis weight (mg) from 12-week old wild-type (110.6±10.4 mg, *n* = 12) and *Alkbh1^−/−^* (85.6±8.4 mg, *n* = 20) males, and 12-month old wild-type (114.4±8.9 mg, *n* = 6) and *Alkbh1^−/−^* (74.3±12.1 mg, *n* = 24) males. (B) TUNEL staining (middle panel) and DAPI staining (left panel) of testis sections from 9-month old wild-type and *Alkbh1^−/−^* mice, showing apoptosis in *Alkbh1^−/−^* round (arrowhead) and elongating (arrow) spermatids, and in degenerated germ cells in the subbasal layers of the tubules (middle and right panel). Closer view of one apoptotic elongating spermatid is shown in the lower panels. (C) Double immunostaining of XY-bodies in mid-pachytene cells. Testicular cells from 12-month old wild-type and *Alkbh1^−/−^* males were spread and stained with two different markers against XY-bodies. MacroH2A (green), FK2 (red) and DAPI (blue). (Magnifications: (B) ×20, (C) ×20).

Reduced testis weight was observed in *Alkbh1^−/−^* males at 12-week and 12-month of age, constituting three-quarters and two-thirds the mean weight of testis from wild-type littermates, respectively (Fig. 4A). TUNEL staining of testes from 12-week old wild-type and *Alkbh1^−/−^* males were histologically indistinguishable and showed no apoptotic cells (data not shown). On the other hand, extensive apoptosis and reduced number of germ cells were revealed in 5–10% of the seminiferous tubules in 9-month old *Alkbh1^−/−^* males (Fig. 4B, Fig. S2). In *Alkbh1^−/−^* testes, no apoptosis was detected in the spermatogonia (Sg) located at the edges of the tubules and in the meiotic spermatocytes (Sc) residing mostly in the two to three subbasal layers (Fig. 4B, Fig. S2). However, numerous apoptotic and degraded cells were seen in the subbasal regions corresponding to spermatocytes and spermatids, as well as in degenerating round and elongating spermatids (Sd) in the more luminal layers of the tubules (Fig. 4B, Fig. S2). In wild-type, a few apoptotic cells were occasionally located mainly at the basal layer of the seminiferous tubules (Fig. 4B, Fig. S2). To better define the basis for arrest in germ cells and the sex-ratio distortion, we focused on the XY-body in the pachytene stage of meiosis. The XY-body is a condensed chromatin structure containing the sex chromosomes, which is thought to be essential for meiotic progression. In mid-pachynema the XY-body forms a spherical structure near the nuclear periphery [25]. Two different markers against XY-bodies were used, macroH2A and FK2, however visible sex-bodies were readily identified in pachytene spermatocytes from 12-month old wild-type and *Alkbh1^−/−^* testes (Fig. 4C). MacroH2A recognizes the sex chromatin, and FK2 detects the abundant ubiquitination of H2A in the XY-body. We also did antibody staining against several specific stages throughout spermatogenesis, but no significant differences between wild-type and *Alkbh1^−/−^* mice were revealed (Fig. S3). The fact that sex-body formation is not impaired in *Alkbh1*-null males does not exclude the hypothesis of an epigenetic and silencing defect of the paternal X chromosome in those mice. Another possibility is that the skewing of the sexes in *Alkbh1^−/−^* mice is related to autosomal sex-ratio distortion. It is well known that most mechanisms that affect segregation distortion act in the male gametes and affect male fertility [24].

### Expression Profiling in Wild-Type and *Alkbh1^−/−^* Testis

**Figure 5:**
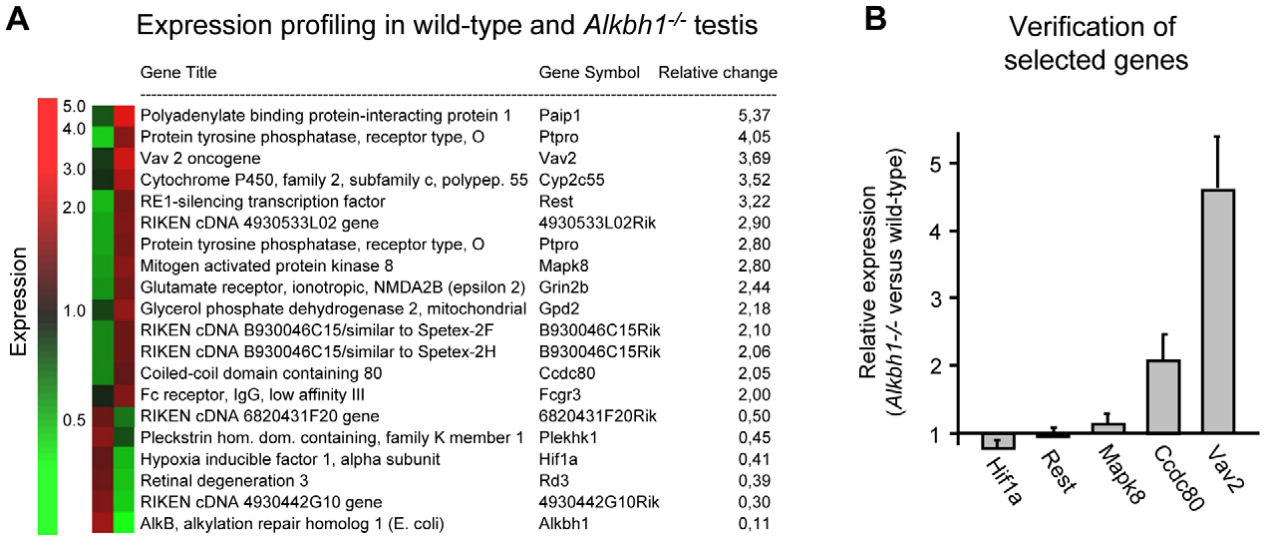
Expression profiling in wild-type and *Alkbh1^−/−^* adult testis. (A) Microarray analysis of whole testes from three wild-type and three *Alkbh1^−/−^* 12-week old males. Results are presented following class comparison analysis and visualized by GeneSpring v 6.0. (B) Verification of differentially expressed genes from the microarray analysis of wild-type and *Alkbh1^−/−^* testes. A selection of genes identified in the class comparison analysis (*Vav2*, *Mapk8*, *Ccdc80*, *Rest*, *Hif1a*) were checked for significance by qPCR. Upregulation of *Vav2* and *Ccdc80* were confirmed, while the differential expression of *Mapk8*, *Rest* and *Hif1a* were not found to be significant. On the RNA used for the microarray study. Reference sample, wild-type *Ccdc80* (RQ = 1.00); endogenous control, *18s*.

Due to the pivotal role of Alkbh1 in mouse survival and potentially in germ cells, we searched for Alkbh1-regulated genes in adult testes. Microarray analysis of whole testes from 12-week old males identified 25 genes that were differentially expressed in *Alkbh1^−/−^* versus wild-type, using the class comparison strategy (Fig. 5A). *Ptpro* were also statistically significantly upregulated in *Alkbh1^−/−^* testes (Table S1; All data is deposited in GEO, accession number GSE22073). The function of PTPRO in adult testis has not been explored, but Avraham et al found expression of PTPRO in testis in humans [26]. Ptpro is suggested to be involved in the differentiation and axonogenesis of central and peripheral nervous system neurons, where it is in position to regulate phosphotyrosine levels in intracellular signaling cascades [27]. qPCR was performed on selected genes, to verify the class comparison analysis (Fig. 5B). Upregulation of *Vav2* and *Ccdc80* was confirmed in *Alkbh1^−/−^* versus wild-type whole testes. Vav2 is a guanine nucleotide exchange factor important for the formatin of adherens junctions between Sertoli cells and spermatids in testis, as well as in the formation of synapses in neurons [28]. The function of Ccdc80, also known as steroid sensitive gene 1, has not been studied in testis, but is supposed to be expressed in this organ according to its EST profile in the Unigene database (http://www.ncbi.nlm.nih.gov/UniGene/ESTProfileViewer.cgi?uglist=Mm.181074). Ccdc80 is expressed in human mesenchymal stem cells and mouse embryo cartilage, suggesting a role in skeletogenesis [29]. Together, these findings point towards a role in regulating the expression of genes having diverse functions – in spermatogenesis, in the nervous system and in skeletogenesis, although the genes affected in the microarray analysis are merely indirect targets of the Alkbh1 protein.

### *Alkbh1* Deficiency Causes Unilateral Eye Development

**Figure 6:**
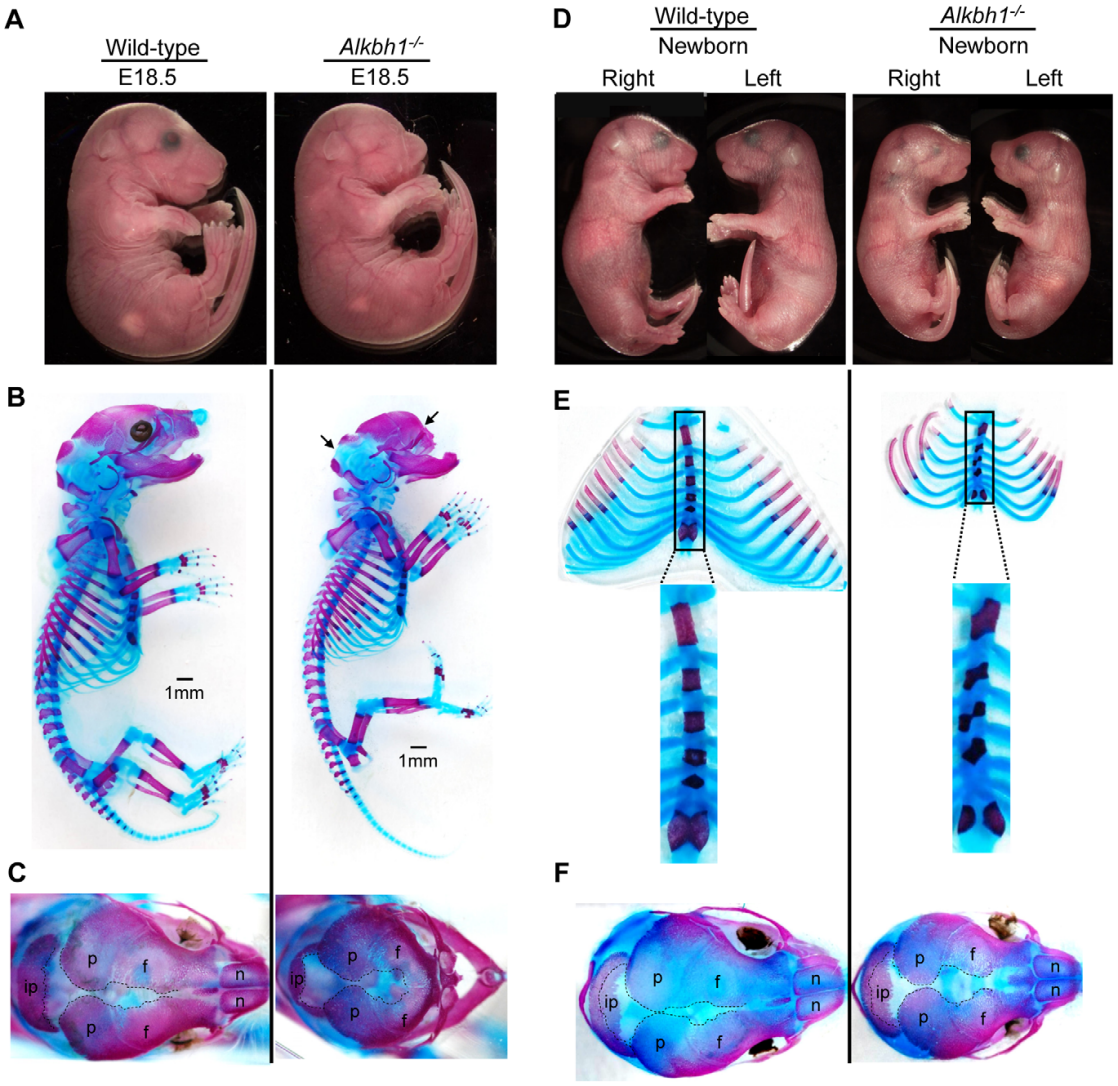
Eye and skeletal phenotype of *Alkbh1^−/−^* embryos and newborns, showing ossified areas in red and cartilage in blue. (A) Side view of embryos at E18.5. The *Alkbh1^−/−^* has a bilateral anophthalmic phenotype, a shortened snout and slightly reduced body size. (B) Skeletal staining of E18.5 embryos displaying missing nasal bones, shortened frontal bones and upward curving of the lower jaws in *Alkbh1^−/−^*. (C) Dorsal view of the craniofacial skeleton of E18.5 embryos showing reduced ossification of the interparietal, parietal and frontal bones leading to bigger sutures in the skull of the *Alkbh1^−/−^*. (D) Side view of newborn mice. The *Alkbh1^−/−^* has a unilateral microphthalmic eye phenotype and reduced body size. (E) Skeletal staining of the rib cage and sternum (in magnification) revealing delayed ossification and severe aberrant fusion of the sternal bands in *Alkbh1^−/−^* newborn mice. Interparietal, ip; parietal, p; frontal, f; nasal, n. (F) Dorsal view of the craniofacial skeleton of newborns demonstrating bigger sutures in the skull of *Alkbh1^−/−^*.

The reduced viability of *Alkbh1* deficient mice and the expression pattern of *Alkbh1* during embryonic development prompted us to analyse embryos and mice at earlier developmental stages. Both *Alkbh1^+/−^* and *Alkbh1^−/−^* mice showed embryonic (E) and postnatal (P) lethality, ranging from E9.5 to P28 (data not shown). Both embryos and neonatal mice clearly displayed an incompletely penetrant defect of small (microphthalmia) or missing (anophthalmia) eyes, and most often in the right eye (unilateral) (Fig. 6A, D). Eye malformations such as microphthalmia and anophthalmia occur in the mouse if eye morphogenesis is disrupted during the critical stages between E9.5 and E13.5 [30]. Small or missing eyes were observed in 18% of *Alkbh1^−/−^* embryos (*n* = 7/39) and 9% of *Alkbh1^+/−^* embryos (*n* = 7/79) at E11.5–E12.5. In surviving adults, eye defects were observed in 9% of *Alkbh1^−/−^* mice (*n* = 14/150) and 0.5% of *Alkbh1^+/−^* mice (*n* = 1/198). Eye defects varied from unilateral (one side) to bilateral (both sides) microphthalmia or anophthalmia, or unilateral microphthalmia in combination with unilateral anophthalmia (Fig. 6A, D). Intriguingly, the disturbed eye development affected the right eye more severely than the left eye, bearing resemblance to the histone arginine demethylase *Jmjd6* and the HMG box factor *Sox3* null phenotypes in mice [31]–[33].

To identify any abnormalities in addition to small or missing eyes, E18.5 embryos and newborn mice were analysed by skeletal staining of bone (Alizarin red) and cartilage (Alcian blue). Multiple defects were detected in the craniofacial, sternum and limb skeleton of mice lacking Alkbh1 (Figs. 6A–F). In the skull, reduced or missing intramembranous ossification resulted in enlarged sutures (Figs. 6B–C, F), while in the sternum, delayed ossification and aberrant fusion of the sternal bands were observed (Fig. 6E). Skeletal staining also showed assymetric shortening of the nasal bones, curving unilaterally in *Alkbh1^−/−^* mice causing mal-developed teeth (Fig. S4A), as well as reduced ossification in the phalanges (P) and the metatarsals (M) of the autopod of *Alkbh1^−/−^* newborns (Fig. S4B). The most crucial step in skeletal morphogenesis is the formation of mesenchymal condensations at E9.5 to E11.5 in mouse development [34]. The *Alkbh1* variable phenotype indicates incomplete condensation of mesenchymal cells during skeletogenesis.

### Incomplete Penetrance of Unilateral Eye Defects

**Figure 7:**
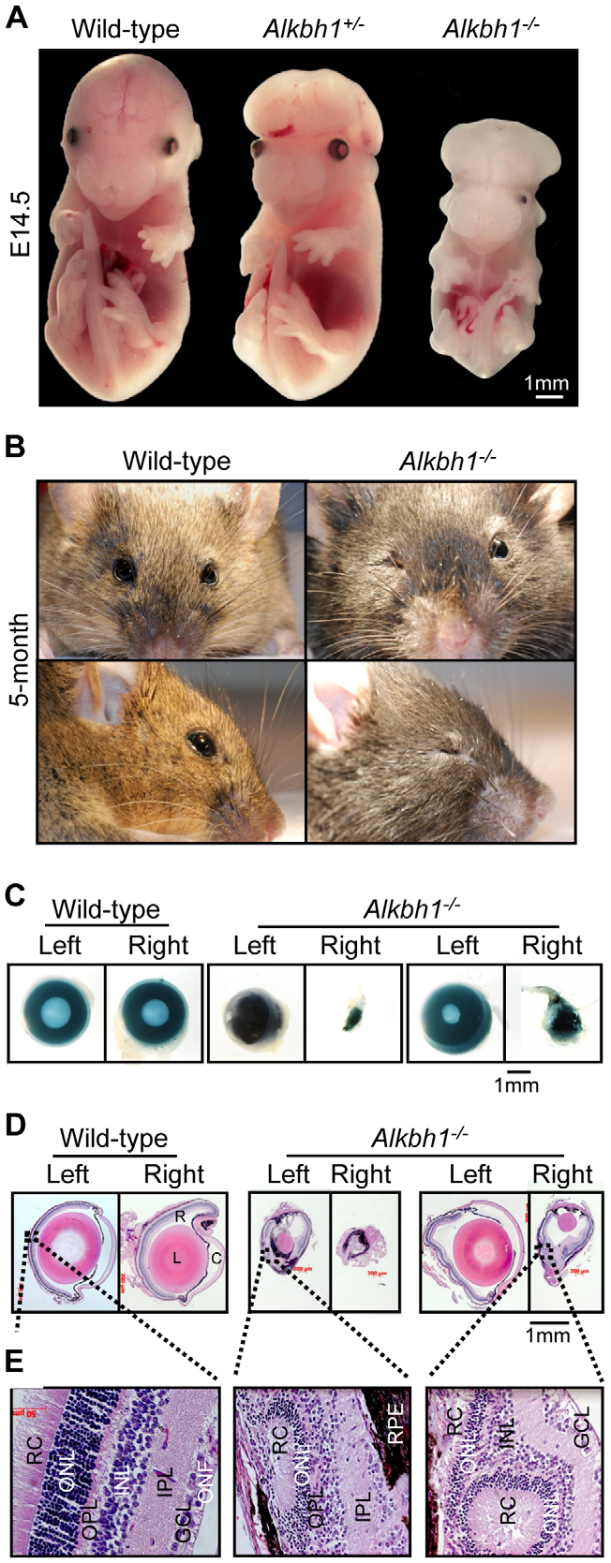
Incomplete penetrance of eye defects and exencephaly of *Alkbh1^−/−^* embryos and adults. (A) Frontal view of wild-type, *Alkbh1^+/−^*, and *Alkbh1^−/−^* embryos at E14.5. The *Alkbh1^+/−^* and *Alkbh1^−/−^* embryos exhibit exencephaly in combination with a shortened, broad snout, while the *Alkbh1^−/−^* embryo also has a bilateral microphthalmic eye phenotype and severely reduced body size. (B) Frontal view and side view of wild-type and *Alkbh1^−/−^* adult mice. The *Alkbh1^−/−^* mouse has a unilateral microphthalmic eye malformation. (C) Whole-mount view of fixated eyes from wild-type and *Alkbh1^−/−^* adult mice demonstrating absent pupils and various degrees of eye malformations. (D) Histological analysis of paraffin-embedded eye sections. In *Alkbh1^−/−^* eyes, the lens is either missing or small and displaced in the eye field. Retinal cells appear degenerated or have lost their organized lamination pattern. R, retina; L, lens; C, cornea. (E) Closer view of the retina shown in d. Neural retinal cells are dysplastic with inclusions of rods and cones surrounded by outer nuclear layer cells. Retinal pigment epithelium cells are found inside the multi-layered neural retina. RC, rods and cones; ONL, outer nuclear layer; OPL, outer plexiform layer; INL, inner nuclear layer; IPL, inner plexiform layer; GCL, ganglion cell layer; ONF, optic nerve fibers. (Magnifications: (D) ×2,5, (E) ×40).

Penetrance is described as incomplete when a trait associated with a specific allele is expressed in a proportion of the population carrying the allele variant [35]. The eye phenotype associated with lack of the *Alkbh1* allele is characterized by incomplete penetrance (Fig. 7A–B). The *Alkbh1^−/−^* mouse in Fig. 7B has developed normally except for the deficiency of one eye. In contrast, the *Alkbh1^−/−^* embryo in Fig. 7A has gross developmental abnormalities, in addition to one small eye with only a residual mass of retinal cells, and one eye missing. The excessive brain tissue outside the skull is characteristic of a condition in which the neural tube fails to close, called exencephaly. Exencephaly is a neural tube defect (NTD), together with spina bifida (open spine) and anencephaly (open skull) [36]. At E10.5–E11.5, NTDs were observed in 23% of *Alkbh1^+/−^* embryos (*n* = 12/52) and 10% of *Alkbh1^−/−^* embryos (*n* = 3/31). The defects originated primarily from disrupted closure in the midbrain-hindbrain region (Fig. 7A) and upper spinal region, and were frequently associated with head and facial malformations (Fig. S4C). Around 50% of embryos with NTDs simultaneously displayed eye malformations (*n* = 14/27). The eye- and NTD-defects observed in *Alkbh1* mutants correspond with the expression pattern of *Alkbh1* seen in embryos and adult mice.

Gross morphological and histological analysis of adult *Alkbh1^−/−^* eyes revealed a range of serious deformities and size variations (Fig. 7C–D). Hematoxylin and eosin (HE) staining of paraffin-embedded sections showed that the lens was either completely missing or clearly smaller and displaced in the eye field (Fig. 7D). Furthermore, the lens fiber cells had lost their ordered lamination pattern, and swollen and liquefied fibers as well as vacuoles were seen throughout the lens (Fig. S4D). In retinal cells, there was a severe loss of organization even though all the retinal cells were present (Fig. 7D). In some areas, the neural retina (NR) was dysplastic with inclusions of rods and cones surrounded by outer nuclear layer cells (ONL), forming rosettes (Fig. 7E). In others, regions of thick layers of retinal pigment epithelium (RPE) cells were observed, with RPE cells appearing inside the NR layers in direct contact with the lens (Fig. 7E). Hence, Alkbh1 is important for growth and appropriate positioning and survival of lens and retinal cells.

### Altered Expression of *Bmp*s in *Alkbh1* Deficient Embryos

**Figure 8:**
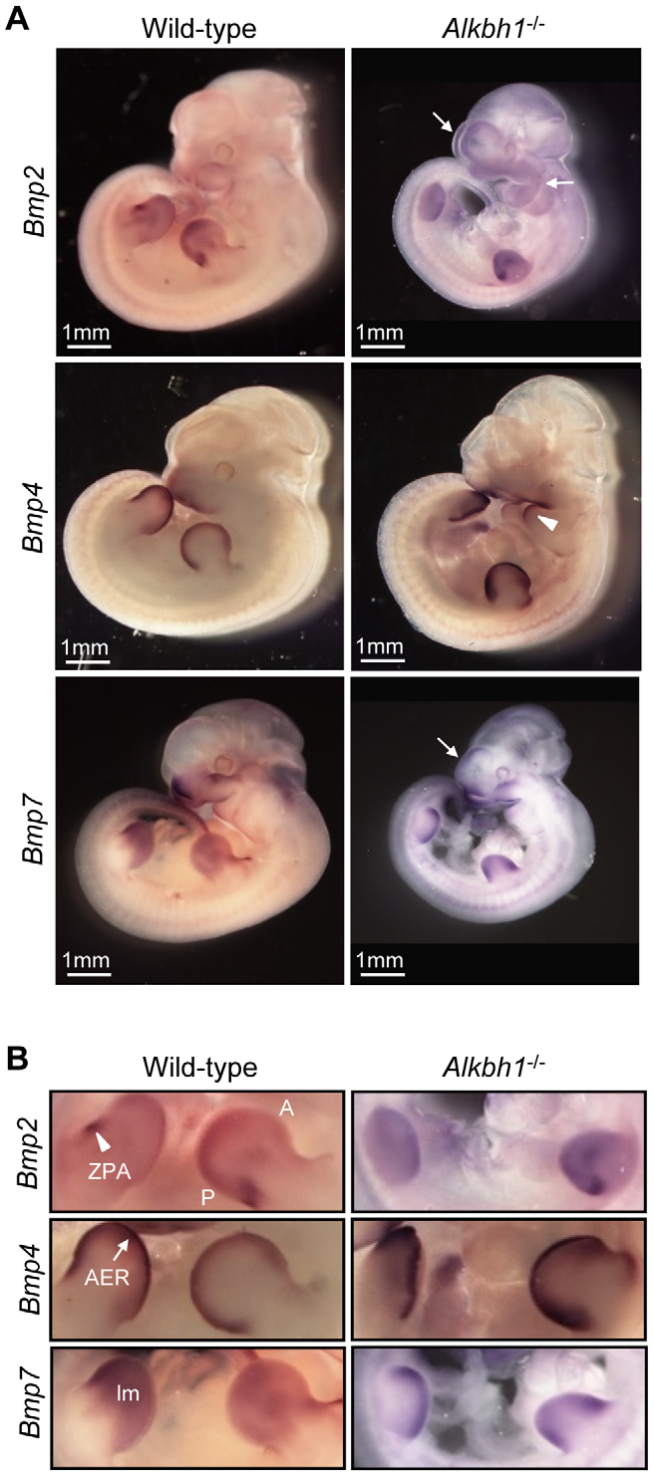
Misexpression of *Bmp2*, *Bmp4* and *Bmp7* in *Alkbh1* deleted embryos at E11.5. (A) Whole-mount *in situ* hybridization of *Bmp2*, *Bmp4* and *Bmp7* in *Alkbh1^−/−^* embryos at E11.5, side view. Altered expression is shown in the frontonasal process, the telencephalon (tc) and in the branchial arches (ba). (B) Closer view of the limbs from the whole-mount *in situ* hybridization of *Bmp2*, *Bmp4* and *Bmp7* in *Alkbh1^−/−^* embryos at E11.5, side view. The expression is altered in the zone of polarizing activity (ZPA), the apical ectodermal ridge (AER) and in the limb mesenchyme (lm). A, anterior; P, posterior.

Embryonic development and tissue regeneration are regulated by four major families of signaling molecules. One of the largest families is the bone morphogenetic proteins (Bmps) [37]. In skeletogenesis, Bmp signaling plays an important role in regulating chondrocyte differentiation and establishment of joint boundaries [38]. Current evidence indicates that Bmp2, Bmp4 and Bmp7 are the main source of Bmp signaling in vertebrate limb buds [39]. Similar signaling mechanisms are suggested for growth and regional specification of the forebrain, branchial arches and eye during development [40]–[42]. This prompted us to examine the expression of *Bmp2*, *Bmp4* and *Bmp7* in apparently normal *Alkbh1^−/−^* embryos at E11.5 (Fig. 8A). *Bmp2* and *Bmp7* were induced in the lateral telencephalon (tc) of *Alkbh1^−/−^* embryos, and expression of *Bmp2* also increased in the frontonasal process (Fig. 8A). Moreover, *Bmp4* and *Bmp7* became upregulated specifically in the maxillary and mandibular cleft, while *Bmp2* was upregulated throughout the maxillary, mandibular and hyoid mesenchyme (Fig. 8A). In limb buds, *Bmp4* and *Bmp7* were highly upregulated in the apical ectodermal ridge (AER) and in two broader domains anteriorly and posteriorly (Fig. 8B). *Bmp2* expression disappeared from the posterior domain in hindlimb, and expression in AER of forelimb diffused proximally into the mesenchyme (lm) (Fig. 8B). The disrupted expression of *Bmp2*, *Bmp4* and *Bmp7* might be the cause of the somewhat smaller limb buds in *Alkbh1^−/−^* embryos. Regulation of these *Bmp* genes is important for AER formation, which is the major signaling center for limb outgrowth [37]. In general, both increased and decreased Bmp signaling can result in skeletal phenotypes [38].

## Discussion

Our data point towards an important role of Alkbh1 in spermatogenesis and embryonic development. Several genes involved in spermatogenesis, in the nervous system and in skeletogenesis were found to be differentially expressed in *Alkbh1^−/−^* whole testes. Adult males deficient in *Alkbh1* exhibited dramatically increased levels of apoptosis in 5–10% of the seminiferous tubules of testes; in spermatids and in degenerated germ cells in the subbasal regions corresponding to spermatocytes and spermatids. The reduced number of all spermatogenic cells in the apoptotic tubules, might reflect an indirect effect of prolonged arrest in spermatids in the affected tubules. Similar nonspecific defects have been seen in *miwi*-null mice [43] and TRF2 mutants [44], [45]. Most genes involved in spermatogenesis display pleiotropic and leaky mutant phenotypes, as presented in this paper. Targeted disruptions of genes resulting in a variable range of defects and incomplete penetrance of spermatogenesis is even the case for regulatory genes, such as those encoding RNA binding proteins DAZLA [46] and MVH [47], and cell cycle regulators HSP-70.2 [48], [49] and cyclin A1 [50].

The sex-ratio distortion lead us to study the XY-bodies in pachytene spermatocytes from *Alkbh1^−/−^* testes, however visible XY-bodies were detected showing that X and Y chromosomes paired normally during male meiosis. This does not exclude the hypothesis of an epigenetic and silencing defect of the paternal X chromosome in those mice, which could explain the sex-ratio distortion observed. Moreover, embryonic and postnathal lethality seen in *Alkbh1^−/−^* mice seem to be of paternal origin and *Alkbh1^−/−^* males exhibit subfertility compared to wild-type males. Several characteristics of the *Alkbh1^−/−^* mice are comparable with those described for the *Jmjd1a* histone lysine demethylase and the *G9a* histone lysine methyltransferase mutant mice, although to a milder extent than demonstrated in the histone disrupted mouse models [51], [52]. *Jmjd1a* deficiency caused extensive germ cell apoptosis and blocked spermatid elongation, resulting in small testes and infertility in male mice [51]. Inactivation of *G9a* in the germ-lineage resulted in sterility due to a drastic loss of mature gametes [52]. The specific upregulation of *Alkbh1* in the pachytene stage, together with the sex-ratio distortion, suggests a potential to regulate the expression of genes during meiosis in the germline. Future investigations will focus on the regulation of specific genes in pachytene spermatocytes isolated from *Alkbh1^−/−^* and wild-type testes.

*Alkbh1* mutant mice displayed phenotypes of incomplete penetrance, including unilateral eye malformations, neural tube defects, and craniofacial and skeleton associated abnormalities. Around 10% of the *Alkbh1^−/−^* mice appeared relatively normal, whereas the most affected mice died early during embryogenesis. The phenotypes are similar to published results on the bone morphogenetic proteins (Bmps), such as haploinsufficiency of Bmp2 causing exencephaly comparable to Fig. 7A [53], and compound heterozygous mutants for Bmp2 and Bmp4 showing unilateral microphthalmia similar to Fig. 6–7 [54]. In addition, postnatal lethality and sex-ratio distortion against females have been shown in *Bmp4^tm1/+^* heterozygous at weaning [55]. Altogether, this led us to investigate the effect on Bmps, and the misexpression of *Bmp2*, *Bmp4* and *Bmp7* in *Alkbh1^−/−^* embryos at E11.5 might explain the inconsistent phenotypes presented. This is due to the critical dependence of gene dosage for proper Bmp function together with the expression- and function-overlap of the Bmps in different tissues [39], [40]. Mouse models of Bmp4 and Bmp7 have shown that redundancy between Bmp4 and Bmp7 is not sufficient to prevent the eye phenotype to occur [40], [41], [56]. In the skull, signaling pathways involving Bmp2, Bmp4 and Bmp7 regulate mesenchymal condensation size, and intense expression of these signaling genes is necessary for closure of sutures [34]. In addition to modifier genes such as Bmps, genetic and epigenetic components can cause variable phenotypic outcomes from specific genes [57], leading to irregular patterns of inheritance as seen for the *Alkbh1* deficient mice. A recent paper has shown that the osteoblast-specific transcription factor Osterix is regulated by the JmjC histone demethylase NO66 [58]. Experiments in the chick embryo have revealed that epigenetic factors are required for the establishment of left-right asymmetries, together with the action of well-studied genetic and signaling mechanisms [59], [60].

The reduced viability and developmental phenotypes apparent in our mouse model, was not reported in the *Alkbh1*-null mice generated by Pan et al [20]. However, they showed severe growth defects in *Alkbh1^−/−^* embryos and newborns in addition to placentas [20], and the growth retardation demonstrated in pups at four weeks of age are comparable with our data (Pan et al. Suppl. Fig. 2 and this paper Fig. S1). No obvious color variation (from red/pink to pale brown/bluish) or growth retardation was observed in *Alkbh1^−/−^* placentas compared to wild-type placentas. Our results are based on extensive breeding studies of *Alkbh1* targeted mice, which revealed a dramatic effect on lethality and sex-ratio in adult mice. We therefore sought to characterize testes and embryos in more detail, as well as the prominent abnormalities in eye development. The different mouse background chosen as well as the dissimilar targeting strategies deleting different parts of the *Alkbh1* gene (Exon 6 in our strain, Exon 3 in Pan et al) could be a possible explanation for the discrepancies in the penetrance of phenotypes in the two knockout mice models. Even so, together with the findings on Alkbh1 by Pan et al, these data suggest that the effect of *Alkbh1* deficiency is pleiotropic and dependent on cell type and/or stage of development.

Recent studies have recognized roles for 2-oxoglutarate dependent dioxygenases in histone and nucleic acid demethylation, as well as in signaling protein hydroxylation [19]. For the demethylating enzymes, several have been shown to carry out its reaction in a manner similar to the potential Alkbh1 mediated, iron- and 2-oxoglutarate dependent, hydroxylation [1], [2], [61]. Previously, mouse models for histone methyl transferases and histone demethylases have been characterized with multiple developmental defects [31]–[33], [62]. Our working hypothesis, based on the variable developmental phenotype of *Alkbh1* deficient mice together with the localization of Alkbh1 to nuclear euchromatin [20], is that Alkbh1 possibly works as a histone demethylase during embryogenesis and spermatogenesis. We believe that the hydroxylation activity of Alkbh1 is dependent on yet undefined partners specific for the different stages/tissues where it has an important role, and this will be addressed in future studies for the pachytene stage of meiosis in male germ cells – when homologues chromosomes pair and crossing over can occur.

## Supporting Information

Figure S1 Average body weight of *Alkbh1* targeted males and females. (A) 1-month old wild-type (19.0±2.0 g, *n* = 45) and *Alkbh1^−/−^* (14.6±3.8 g, *n* = 48) males, and 1-month old wild-type (17.7±1.7 g, *n* = 43) and *Alkbh1^−/−^* (14.8±2.2 g, *n* = 33) females. The average weight was 25% lower for *Alkbh1^−/−^* males than for wild-type males and 15% lower for *Alkbh1^−/−^* females than for wild-type females. About one out of five *Alkbh1^−/−^* males showed more than 40% lower weight compared to wild-type males. (B) 9-month old wild-type (40.5±4.2 g, *n* = 23) and *Alkbh1^−/−^* (32.5±2.9 g, *n* = 31) males, and 9-month old wild-type (31.5±3.4 g, *n* = 28) and *Alkbh1^−/−^* (29.3±3.8 g, *n* = 41) females. The average weight of *Alkbh1^−/−^* males was 20% below that of wild-type males, and the average weight of *Alkbh1^−/−^* females was 7% below that of wild-type females. No weight difference was demonstrated between the *Alkbh1^+/−^* and wild-type (data not shown). +/+ (wild-type), black bars; −/− (*Alkbh1^−/−^*), grey bars.
(0.10 MB TIF)

Figure S2 Closer view of the DAPI and TUNEL staining of testis sections shown in Fig. 4. (A, B) Sections from 9-month old wild-type (left panel) and *Alkbh1^−/−^* (right panel) mice are presented. Apoptosis was detected in degenerating spermatids (Sd) in the luminal layers of *Alkbh1^−/−^* tubules, as well as in severely degraded cells in the subbasal regions corresponding to spermatocytes and spermatids. No apoptotic cells were seen in spermatogonia (Sg) and spermatocytes (Sc) in *Alkbh1^−/−^* mice, although the amount of all spermatogenic cells are reduced in the apoptotic tubules. (Magnification: ×20).
(3.93 MB TIF)

Figure S3 Immunostaining with stage-specific antibodies against spermatogenic cells in *Alkbh1* deficient testes. (A) Testis sections from 12-month old wild-type and *Alkbh1^−/−^* males stained with TRA98 antibody specific for spermatogonia, which were present both in wild-type and mutant. Although several tubules showed spermatogonia not only in the first basal layer, but also in the subbasal layers in the *Alkbh1^−/−^* mice, no significant differences were detected when compared to wild-type. (B) Testis sections from 12-month old wild-type and *Alkbh1^−/−^* males stained with TRA369 specific for pachytene spermatocytes through elongating spermatids, which were present both in wild-type and mutant. (Magnification: ×20).
(1.84 MB TIF)

Figure S4 Skeletal defects, eye defects in combination with NTD, and lens defects in *Alkbh1* targeted mice. (A) Craniofacial defects. Dorsal view of the craniofacial skeleton of adult mice showing assymetric shortening of the nasal bones, curving unilaterally in *Alkbh1^−/−^* mice causing mal-developed teeth (*n* = 4 *Alkbh1^−/−^*; *n* = 1 *Alkbh1^+/−^*). Ossified areas are shown in red and cartilage in blue. (B) Limb defects. Dorsal view of the autopod limb skeleton revealing reduced ossification in the phalanges (P) and the metatarsals (M) of the autopod of *Alkbh1^−/−^* newborns (*n* = 4/4 *Alkbh1^−/−^*). Ossified areas are shown in black and cartilage in blue. (C) Eye defects and NTDs. Side view of embryos at E12.5. The *Alkbh1^−/−^* embryo has a bilateral microphthalmic eye phenotype in combination with a neural tube defect (NTD). The NTD is originating from disrupted closure in the upper spinal region, and is associated with head and facial malformations leading to a shortened, broad snout. In addition, a severe intracranial hemorrhage is visible. (D) Lens defects. Histological analysis of paraffin-embedded eye sections from adult mice. In *Alkbh1^−/−^* eyes the lens fiber cells have lost their ordered lamination pattern, and swollen and liquefied fibers as well as vacuoles are seen throughout the lens. (Magnification: ×10).
(6.28 MB TIF)

Table S1 Statistically upregulated genes in *Alkbh1^−/−^* versus wild-type testes identified in the microarray analysis. Microarray analysis of RNA extracted from whole testes from three wild-type and three *Alkbh1^−/−^* 12-week old males identified 6 genes that were statistically upregulated in *Alkbh1^−/−^* versus wild-type. To find differentially expressed genes, t-test with randomized variance was used as statistical test and the cut-off (p-value) was set to 0.05 with a FDR correction.
(0.22 MB TIF)
