# Supplementary figures and images for: Mice Lacking Alkbh1 Display Sex-Ratio Distortion and Unilateral Eye Defects
Source: PLoS One. 2010 Nov 3;5(11):e13827. doi: 10.1371/journal.pone.0013827 (PMC2972218; doi:10.1371/journal.pone.0013827)

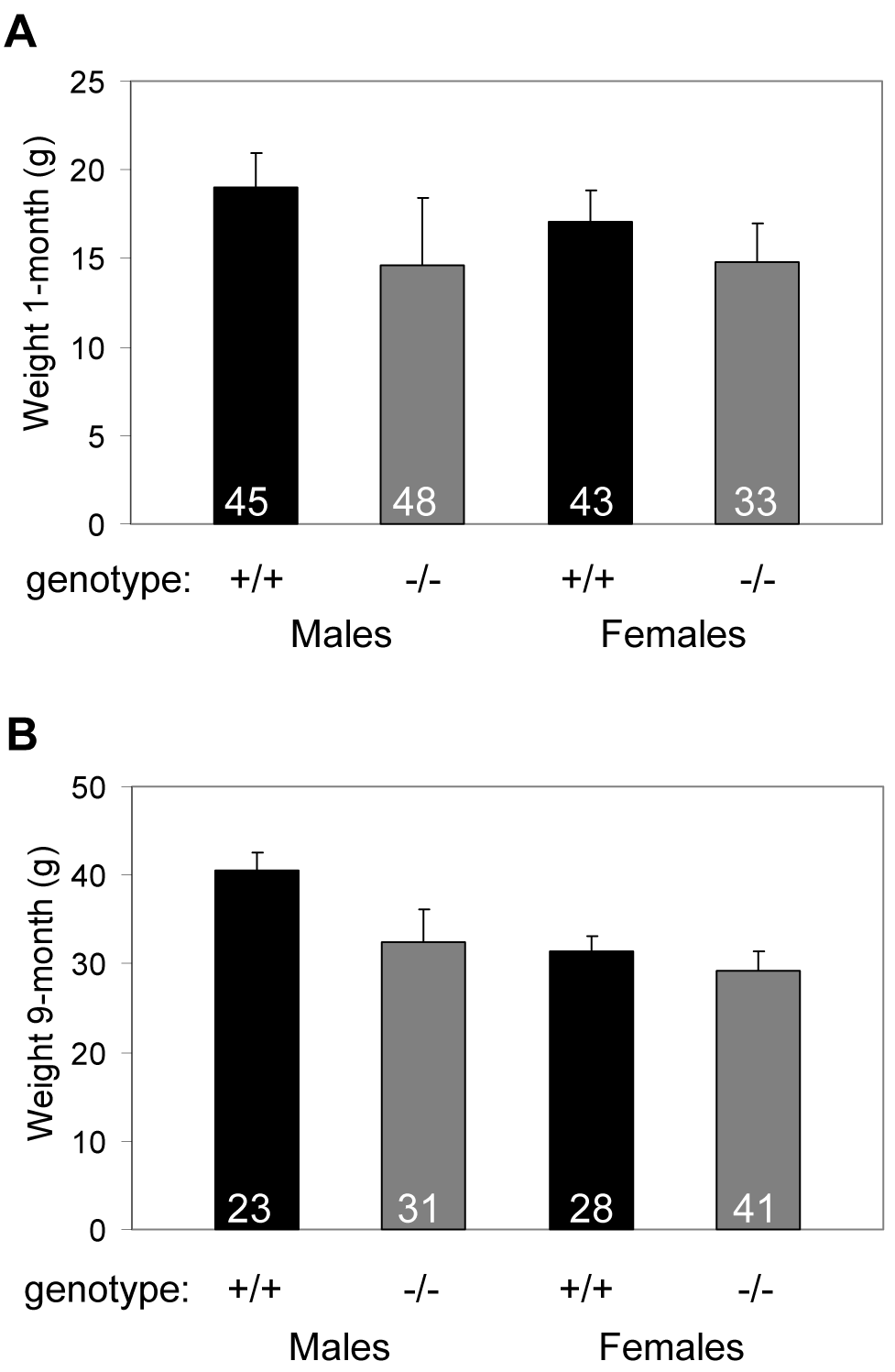

Supplement: Figure S1 — Average body weight of Alkbh1 targeted males and females. (A) 1-month old wild-type (19.0±2.0 g, n = 45) and Alkbh1−/− (14.6±3.8 g, n = 48) males, and 1-month old wild-type (17.7±1.7 g, n = 43) and Alkbh1−/− (14.8±2.2 g, n = 33) females. The average weight was 25% lower for Alkbh1−/− males than for wild-type males and 15% lower for Alkbh1−/− females than for wild-type females. About one out of five Alkbh1−/− males showed more than 40% lower weight compared to wild-type males. (B) 9-month old wild-type (40.5±4.2 g, n = 23) and Alkbh1−/− (32.5±2.9 g, n = 31) males, and 9-month old wild-type (31.5±3.4 g, n = 28) and Alkbh1−/− (29.3±3.8 g, n = 41) females. The average weight of Alkbh1−/− males was 20% below that of wild-type males, and the average weight of Alkbh1−/− females was 7% below that of wild-type females. No weight difference was demonstrated between the Alkbh1+/− and wild-type (data not shown). +/+ (wild-type), black bars; −/− (Alkbh1−/−), grey bars. (0.10 MB TIF) [file pone.0013827.s001.tif]

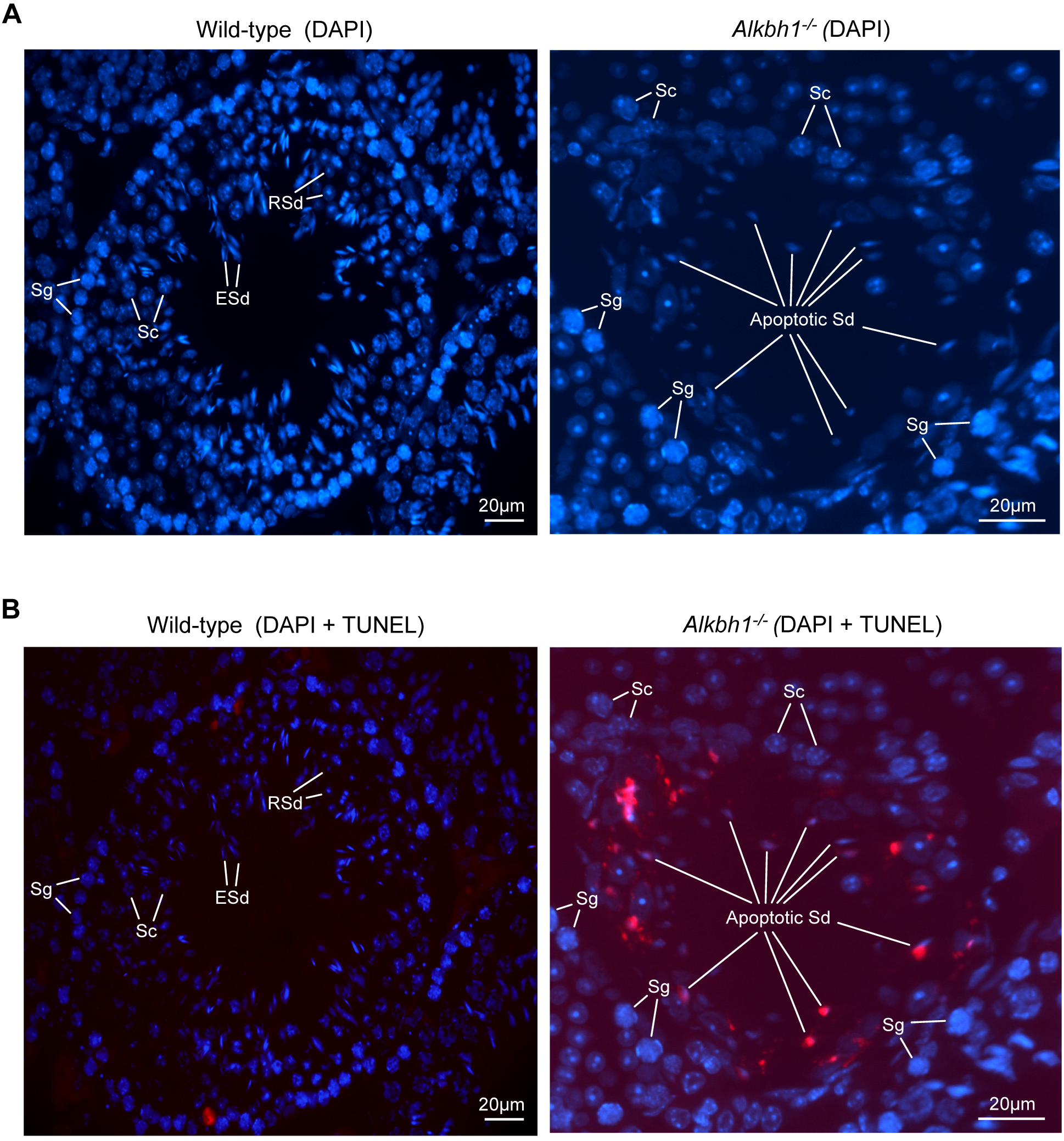

Supplement: Figure S2 — Closer view of the DAPI and TUNEL staining of testis sections shown in Fig. 4. (A, B) Sections from 9-month old wild-type (left panel) and Alkbh1−/− (right panel) mice are presented. Apoptosis was detected in degenerating spermatids (Sd) in the luminal layers of Alkbh1−/− tubules, as well as in severely degraded cells in the subbasal regions corresponding to spermatocytes and spermatids. No apoptotic cells were seen in spermatogonia (Sg) and spermatocytes (Sc) in Alkbh1−/− mice, although the amount of all spermatogenic cells are reduced in the apoptotic tubules. (Magnification: ×20). (3.93 MB TIF) [file pone.0013827.s002.tif]

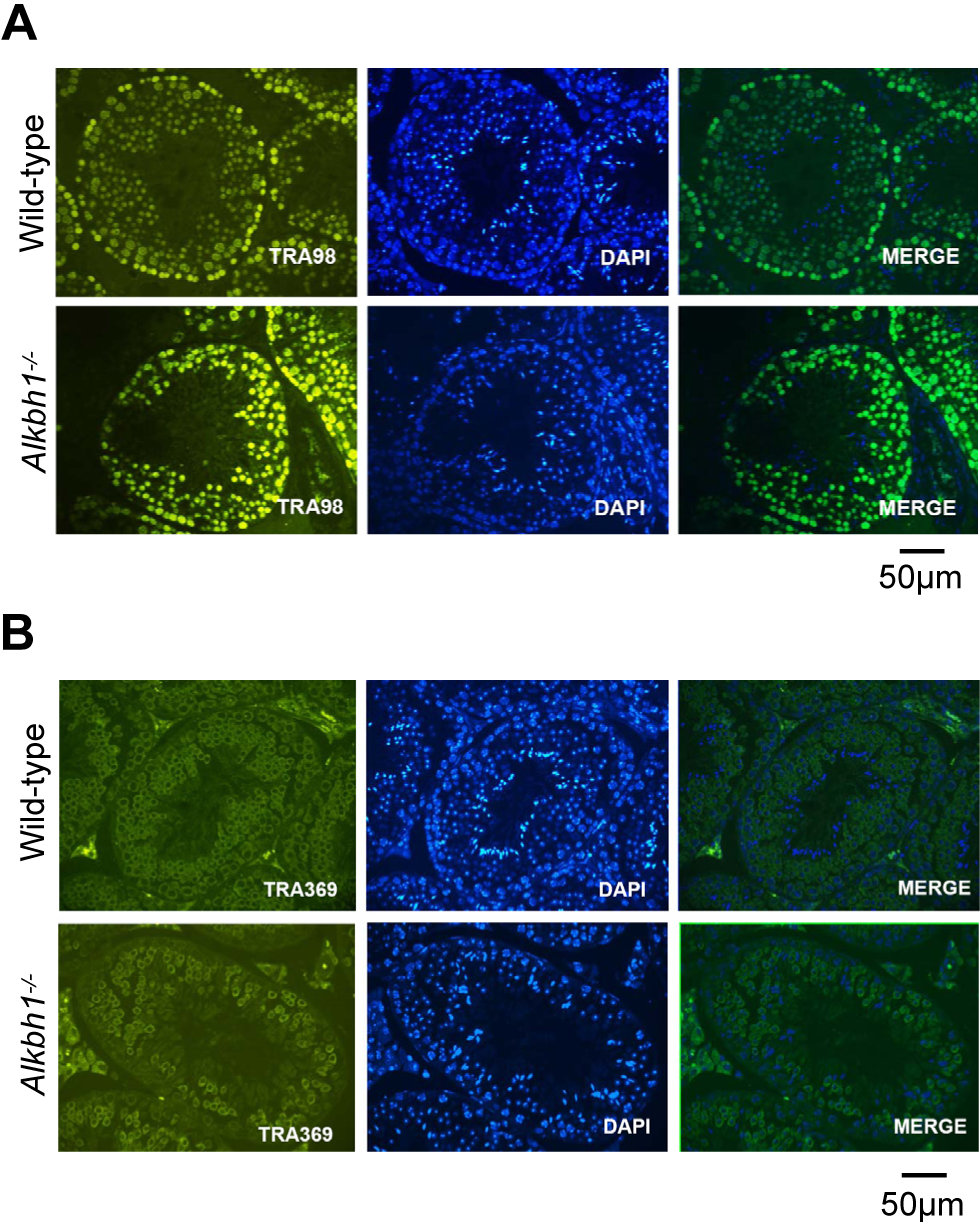

Supplement: Figure S3 — Immunostaining with stage-specific antibodies against spermatogenic cells in Alkbh1 deficient testes. (A) Testis sections from 12-month old wild-type and Alkbh1−/− males stained with TRA98 antibody specific for spermatogonia, which were present both in wild-type and mutant. Although several tubules showed spermatogonia not only in the first basal layer, but also in the subbasal layers in the Alkbh1−/− mice, no significant differences were detected when compared to wild-type. (B) Testis sections from 12-month old wild-type and Alkbh1−/− males stained with TRA369 specific for pachytene spermatocytes through elongating spermatids, which were present both in wild-type and mutant. (Magnification: ×20). (1.84 MB TIF) [file pone.0013827.s003.tif]

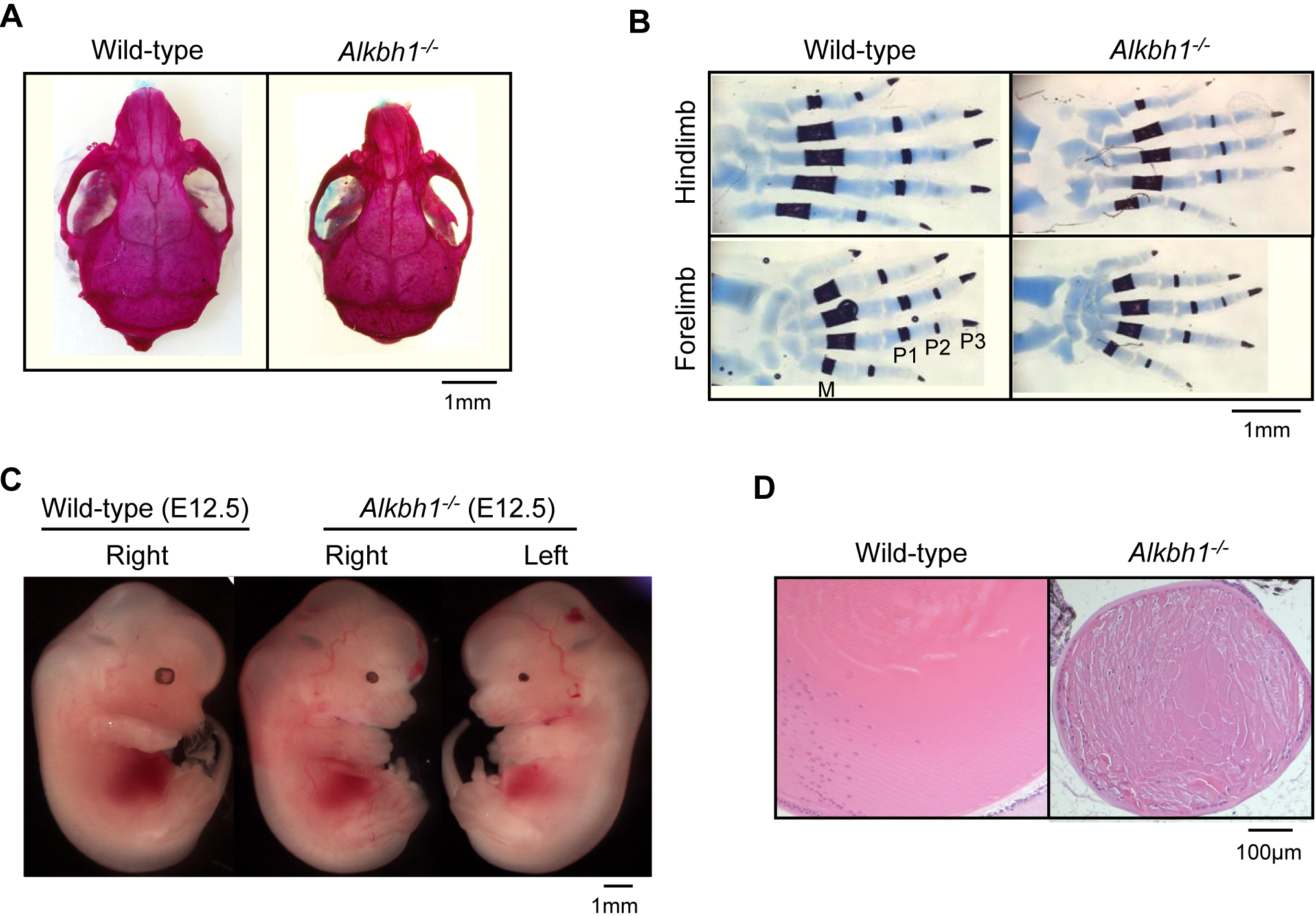

Supplement: Figure S4 — Skeletal defects, eye defects in combination with NTD, and lens defects in Alkbh1 targeted mice. (A) Craniofacial defects. Dorsal view of the craniofacial skeleton of adult mice showing assymetric shortening of the nasal bones, curving unilaterally in Alkbh1−/− mice causing mal-developed teeth (n = 4 Alkbh1−/−; n = 1 Alkbh1+/−). Ossified areas are shown in red and cartilage in blue. (B) Limb defects. Dorsal view of the autopod limb skeleton revealing reduced ossification in the phalanges (P) and the metatarsals (M) of the autopod of Alkbh1−/− newborns (n = 4/4 Alkbh1−/−). Ossified areas are shown in black and cartilage in blue. (C) Eye defects and NTDs. Side view of embryos at E12.5. The Alkbh1−/− embryo has a bilateral microphthalmic eye phenotype in combination with a neural tube defect (NTD). The NTD is originating from disrupted closure in the upper spinal region, and is associated with head and facial malformations leading to a shortened, broad snout. In addition, a severe intracranial hemorrhage is visible. (D) Lens defects. Histological analysis of paraffin-embedded eye sections from adult mice. In Alkbh1−/− eyes the lens fiber cells have lost their ordered lamination pattern, and swollen and liquefied fibers as well as vacuoles are seen throughout the lens. (Magnification: ×10). (6.28 MB TIF) [file pone.0013827.s004.tif]

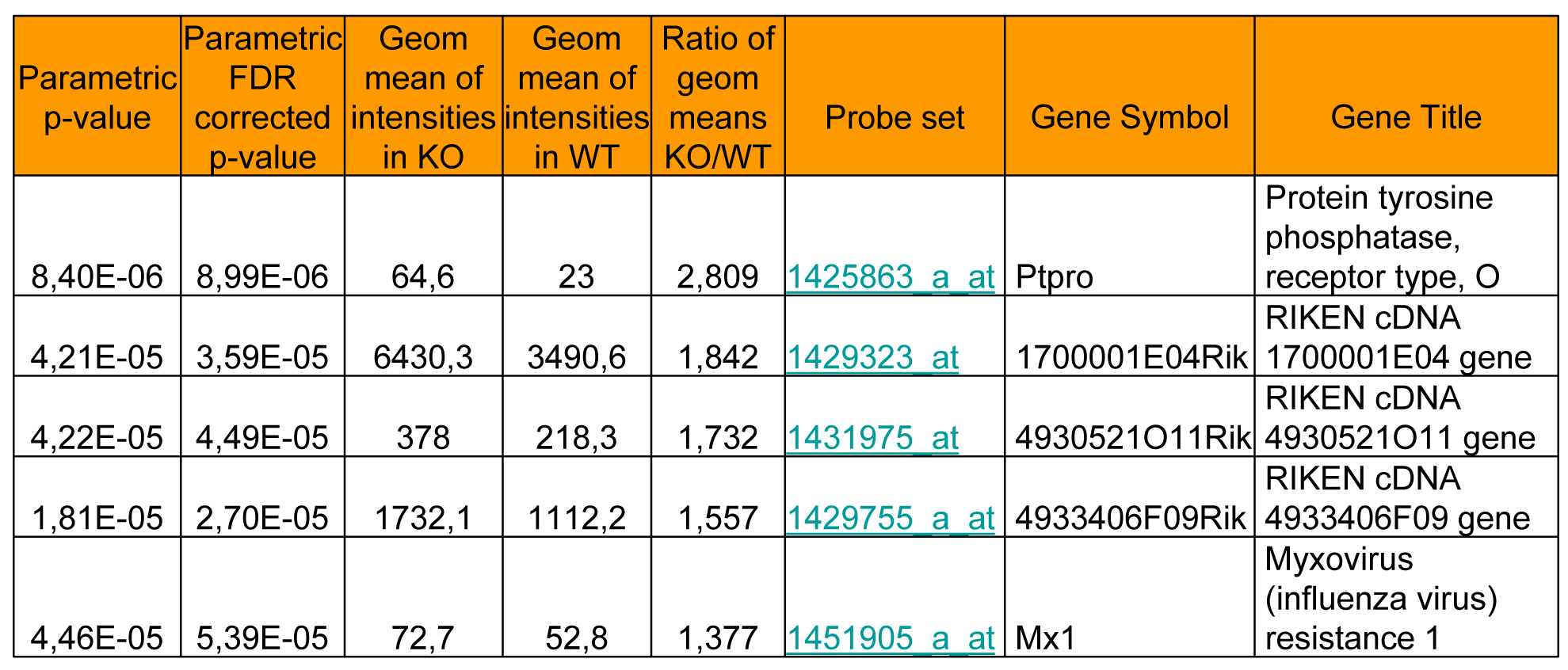

Supplement: Table S1 — Statistically upregulated genes in Alkbh1−/− versus wild-type testes identified in the microarray analysis. Microarray analysis of RNA extracted from whole testes from three wild-type and three Alkbh1−/− 12-week old males identified 6 genes that were statistically upregulated in Alkbh1−/− versus wild-type. To find differentially expressed genes, t-test with randomized variance was used as statistical test and the cut-off (p-value) was set to 0.05 with a FDR correction. (0.22 MB TIF) [file pone.0013827.s005.tif]
